# Locked-in and living delta pathways in the Anthropocene

**DOI:** 10.1038/s41598-020-76304-x

**Published:** 2020-11-11

**Authors:** Maria J. Santos, Stefan C. Dekker

**Affiliations:** 1 University Research Priority Program in Global Change and Biodiversity and Department of Geography, University of Zurich, Zürich, Switzerland; 2 Copernicus Institute of Sustainable Development, Utrecht University, Utrecht, The Netherlands

**Keywords:** Environmental impact, Sustainability

## Abstract

Delta systems are fundamental to the persistence of large human populations, food systems and ecosystem processes. Structural changes in natural and social components of deltas, emerging from past land-use changes, have led deltas to become locked-in loosing the ability to transform back into living deltas, and making them more at risk. We propose a framework to assess whether deltas become locked-in by changes in natural or social infrastructure, by examining the dynamic coupling between population and land-use development over 300 years for 48 deltas globally. We find that 46% of the deltas are defined as living, where population, irrigation, and cropland are correlated. Of the 54% locked-in deltas, 21% show changes in natural infrastructure to cropland (n = 6) or irrigation (n = 4), and 33% (n = 16) show changes in social infrastructure. Most locked-in deltas are in Europe but also in other continents due to decoupled development of population and cropland. While, locked-in deltas due to changes in natural infrastructure have highest average risks, those with changes in social infrastructure and the living deltas have highest risks from future relative sea level rise. These results show that deltas have varying natural and social components derived from a 300 years historical perspective, which are not taken into account in risk assessments for global deltas.

## Introduction

Delta systems are increasingly at risk from global changes. Deltas are fundamental to global sediment dynamics, and face many pressures from both upstream land use change and downstream sea level rise^1–3^. The processes that build and maintain deltas have declined or even halted^4^, with 60% of global deltas’ sediment lost^5^. Currently, 20–30% of global sediment load (4–5 Gt yr^−1^) is intercepted by reservoirs^6^, which will increase with the expansion of hydropower dams^7^ and reservoirs^8^, although sediment fluxes have increased due to deforestation^9^. Delta hydrological systems have ecosystems that depend on sedimentation dynamics^10^. Deltas have lost most of their natural wetlands, which provide them to changing water levels, as well as habitats for many endemic species and in turn aid sedimentation processes necessary for delta dynamics. Delta sediment and water dynamics makes them very fertile and easy to cultivate, making them extremely attractive for human populations; in fact being key locations for the development of many human civilisations, while covering only 0.56% of the area of the world^3^. Disproportionally to their area, deltas provide water and food to large fractions of global populations, which in turn modify delta systems through land use change, land reclamation, channeling, water use and diversion, and added organic inputs and pollution. This poses large risks to deltas as 50% of global delta area is below 8 m a.s.l. and around 24% of delta inhabitants live below 5 m a.s.l.^1–3^. Delta development pathways may become locked-in. We define locked-in if a delta system is unable or too costly to recover to a living delta, resulting in higher risks that those deltas are currently facing^2^. However, it is unclear if and when deltas became locked-in, if lock-in states have infrastructural or social causes that increase their risk. To address this issue, we propose a simple framework that identifies system lock-in by examining the coupling between population and agricultural land use development over the last 300 years for a set of 48 deltas globally. Tessler et al*.*^2^ have used the same 48 deltas to quantify flood risks due to extreme events by using an integrated set of global environmental, geophysical and social indicators.

Deltas strong coupling between their biophysical dynamics and resource use by human populations (water, agriculture, land, etc.;^11,12^) may have created dependencies between these resources. Such dependencies, i.e. lock-ins, have been shown to emerge when, for example, customers are dependent on a vendor for products and services and are unable to use another vendor without incurring substantial switching costs. Similarly, historical development of infrastructure (natural, social) in deltas may create dependencies that cannot be changed without major costs. Deltas like those in Europe are good examples of delta systems that could be locked-in, i.e., where reversing the current system to a natural system is extremely costly, if feasible at all. A typical example is the Dutch Zeeland delta^13^, but similar states are found in the Sacramento-San Joaquin inverse delta^14^, the Mekong delta^15,16^, the Niger delta^17,18^, and the Nile, Yangtze, and Rhine deltas^19^. Such locked-in states may emerge from changes and or removal of natural infrastructure, i.e., river regularization, diking, water diversion, agricultural and urban development^11^. Social locked-in states also emerge from structural conditions of lifestyles, livelihoods, and market incentives, often disregarded when analyzing systems for sustainable outcomes^20^. Further, legacies in the attribution of rights, time lags in regulations and norms (in broad sense institutions) may also lead to social locked-in states^21^. For example, in the United States historical water rights attribution may hinder the design of institutions fit to the current social-environmental components of delta systems^22^. Institutions designed for development and aid, common in developing countries, may also lead to social lock-ins. For example, in Bangladesh, people are strongly connected to their livelihoods due to their strong cultural ecosystem services. Also, the poorest among them for which moving to better places or with greater opportunities is impossible and are therefore unwilling to move despite the strong risks of flooding due to sea level rise^23^. Szabo et al.^24^ clearly demonstrate the link between population demography and delta development for Mekong, Ganges–Brahmaputra and Amazon deltas. These social lock-in costs may result in antitrust action^25,26^, through emerging social unrest due to growing population sizes, unmanaged use of natural resources, and failure to comply with regulations for nature protection. Further, lock-in costs can be exacerbated if locked-in states emerge independently or in tandem in natural and social settings^27^. Our proposed approach considers both the dynamics of natural and social infrastructure changes.

## Locked-in frameworks

While we can understand the factors leading to the emergence of locked-in states in systems, few of the frameworks examining locked-in states have been operationalized at large scales for comparative studies (but see^2,19^). For example, Gerrits and Marks^13^ describe potential mechanisms through which the Dutch Zeeland delta may have become locked-in; two other studies from the Netherlands showcase the emergence of locked-in delta institutions^28^ and built infrastructure^12^. These studies relied on a variety of methods, including: literature reviews of the historical process of delta development^13,15,16^, policy analysis^16,28,29^, interviews^12^, quantitative indicators^19^, and process-based models^30^. While this variety of methods illustrates that lock-in can be detected or inferred from social and natural infrastructural development in deltas, it makes it difficult to compare across deltas and gain a generalizable understanding of the characteristics that lead to emergence of locked-in during delta development.

We propose a novel approach that uses historical land-use development to detect delta locked-in states which accounts for the development of natural and social processes. Land-use development processes emerge from the interplay of changes in population size, demand for natural resources and institutions targeted at their development. We know that land-use change is common in deltas^11^, through land reclamation and development of cropland or irrigated systems. Land-use change to agricultural systems have degraded delta’s natural ecosystems to such a level that reversing these changes would be extremely costly if possible at all, suggesting that this could be an indicator of lock-in. If we consider available land as the resource, it can be used for natural processes, agriculture or urban development until a certain carrying capacity. Over time, the system stays below carrying capacity through technological innovation (i.e. increase efficiency in agricultural production, water management, etc.), lower demand for land (lack of productivity), diverted demand for land to elsewhere (i.e. indirect land use change), or reduced population size (i.e. move out or stop population growth). Alternatively, if carrying capacity is surpassed, then the delta is degraded and the system enters a new pathway. What we need to know then is when to stop delta development before entering a locked-in pathway that leads the system to surpass its carrying capacity and leading to system collapses and state shifts. We posit that if a delta is not locked-in, i.e., a living delta, there should be a positive correlation between development of population density and of agricultural land-uses (cropland and irrigation) because there is still enough land for both to develop. Note that in more modern deltas in rich countries, there might be a land coversion to urban by-passing agriculture and irrigation; nonetheless this is a recent land use transition and the fraction of the delta converted to urban area through this direct land-use change pathway is still very small. Subsequently, a lack of correlation indicates a natural locked-in status due to engineering of natural infrastructure (i.e. land-use conversion from natural ecosystems to cropland and irrigation), and a negative correlation indicates social locked-in due to population and agricultural land-uses developing at odds. Though simple, our approach is justified because we expect that development in deltas is predictable; the closest it gets to the carrying capacity for land the higher the stress into the system. This dynamical approach of driver of historical land use allows for a systematic, robust, transferable and comparable assessment of delta locked-in states at global scale and we will present a test of such method through the analysis of longitudinal panel data over 300 years.

Our objective is to understand to what extent delta lock-in can be detected by looking into the development of land-use in deltas. We first use historical reconstructions to describe the patterns of development of population density globally and in 48 deltas. We used the outputs of the historical database of the global environment HYDE^31,32^ to obtain population density (inhabitants per km^2^) for each delta, as well as the proportion of the delta covered by cropland, irrigation and urban area during the Anthropocene, i.e., 1700 to current.

## Global overview of locked-in deltas

**Figure 1:**
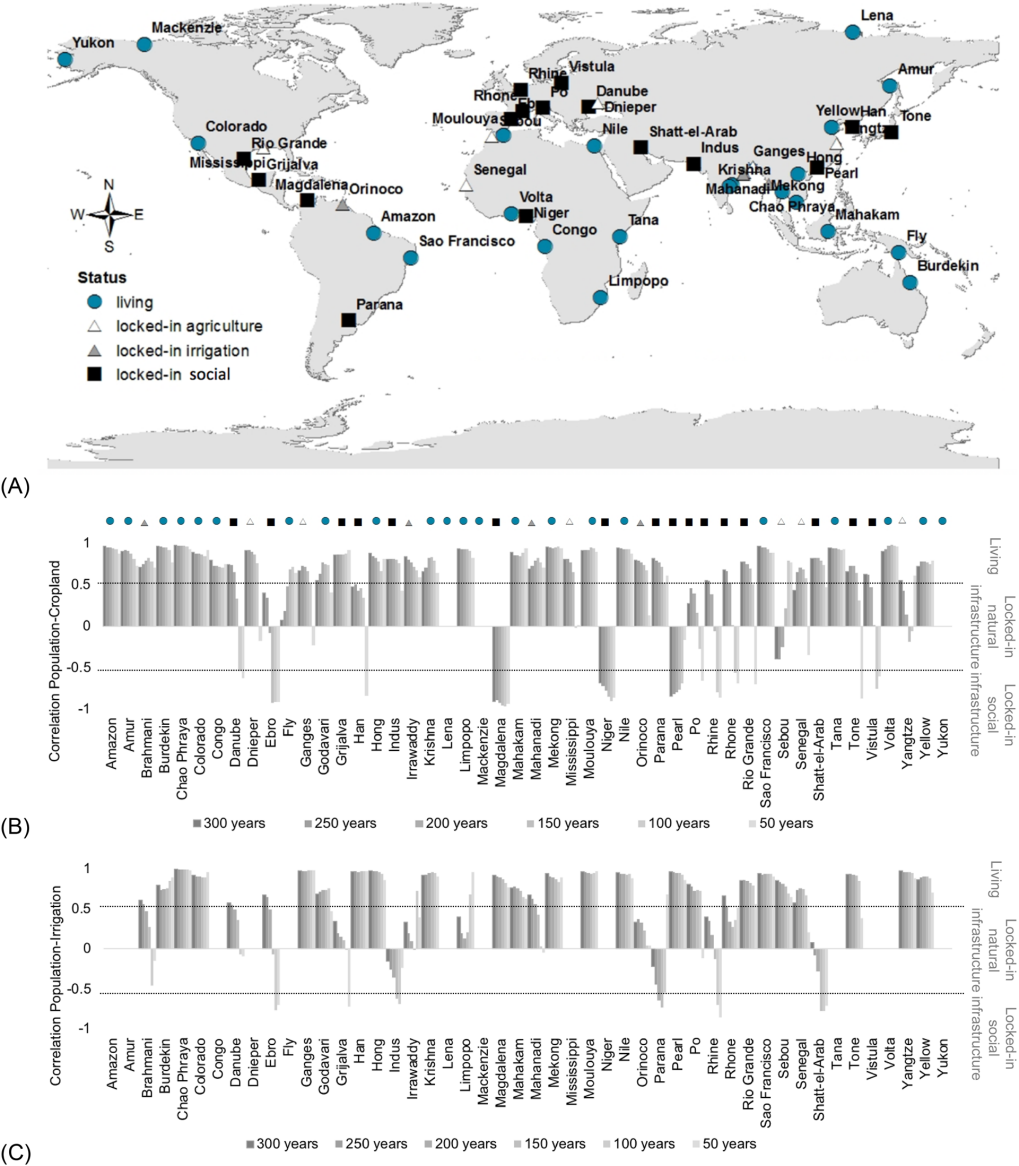
(**A**) Global delta status: living deltas, locked-in deltas due to natural infrastructure development and locked-in deltas due to social infrastructure development. (**B**) and (**C**) development of locked-in over time (bars represent a time period of 50 years, darker grey color represents correlation between population and cropland or population and irrigation in a time span of 300 years, and light grey color represents a time span of the past 50 years; symbols on top of the bars match those in (**A**); for development of the locked-in over different time spans see Figure SM1 in the supplementary material; Figures were produced by the authors using ArcGIS v10 license, ESRI https://www.esri.com/en-us/home).

In total, 54% of the deltas are locked-in, either due to natural or social infrastructure (26 of 48; Fig. 1). Natural locked-in deltas due to engineered infrastructure (i.e. levees, agriculture development, etc.), represent 21% of the deltas (n = 10) from which six deltas shift from positive correlation to no correlation (− 0.5 < r < 0.5) between population density and cropland for at least the past 50 years, and four shift in correlation between population density and irrigation (white and grey triangles in Fig. 1). Deltas like the Dnieper, Ganges, Mississippi, Sebou, Senegal and Yangtze became locked-in due to no further development of cropland, and those like the Brahmani, Irrawaddy, Mahanadi and Orinoco became locked-in due to no further development of irrigation (Figure SM2 in supplementary material). Social locked-in deltas due to institutional infrastructure count for 33% (n = 16) with a negative correlation between population and agricultural use (cropland or irrigation, black squares in Fig. 1 with r < − 0.5) over at least the past 50 years. Some of these deltas, like the Magdalena and Niger, have already been locked-in for the past 300 years (Fig. 1B,C). All European deltas (Rhine, Rhone, Po, Vistula, Danube, Ebro) belong to this locked-in category, mostly due to a negative correlation between population density and cropland. Deltas that show this negative correlation do so mostly because of the lack of cropland development. This could mean that these deltas are likely locked-in because of preferences for urban land-uses and built infrastructure to accommodate their exponentially growing populations. Several of these deltas are located in areas with a long history of human presence and land-use and high land prices (in Europe) which could explain a preference for urban land uses. The Pearl delta also shows a negative correlation between population and cropland. This delta, together with the Yangtze and the Sebou, has reached the highest population densities of our sampled deltas, these large densities need to be accommodated within the available land. Population densities of Pearl and Sebou deltas are twice that of the Ganges, and up to five times higher than for instance the Mekong and Chao Phraya. Eight deltas show no correlation between population development and irrigation, namely the Amazon, Orinoco, Limpopo, Mahakam, Ebro, Danube, Rhine and Rhone. This could be due to growing population and no investment in irrigation. Half of these deltas are located in tropical regions, also exhibiting large population sizes, however water availability is high and irrigation is unneccessary. The other half of these deltas are in Europe. For the Rhone and the Rhine there is no correlation between population and irrigation. These are perhaps the most built and engineered deltas with a long history of infrastructure management. The Ebro and the Danube are in regions that would require irrigation for crop production but where land values for urban uses are higher than crop production gains. The only deltas showing a negative correlation between population density and irrigation are the Grijalva, Indus and Shatt-el-Arab, in regions facing water scarcity.

**Figure 2:**
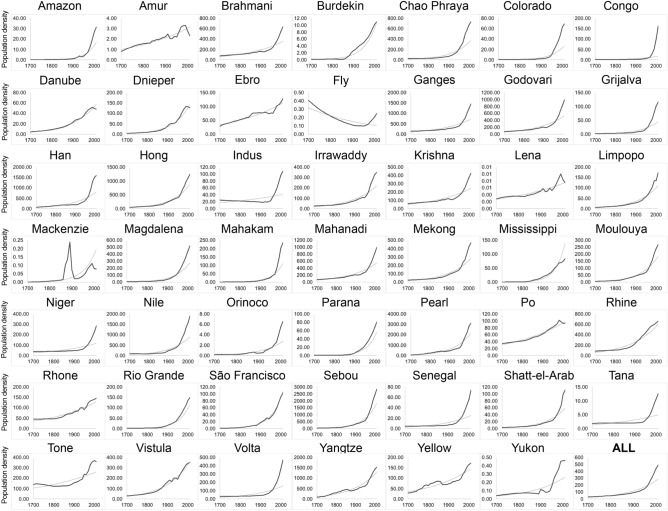
Population development (inh/km^2^) in deltas from 1700 through 2010 (range in the y-axis differs for display purposes). Population development line (solid line) was compared to the line of best fit (dashed line). Equation and goodness-of-fit (coefficient of determination) of the linear regression are shown in Table SM2 in the supplementary material.

We find that 46% of the analyzed deltas are living deltas, i.e., yet to be locked-in (22 out of 48). These deltas are distributed globally, but are found mostly in southern latitudes. Three living deltas show no correlation between population and cropland due to very low densities for both (Lena, Mackenzie and Yukon; Fig. 2 and Figure SM2 in supplementary material), all in northern latitudes. We found the lowest population densities now but also since 1700 between − 50 to 0° latitude in the Southern hemisphere and above 50° latitude in the Northern hemisphere. Most deltas have been living deltas since 1700 (legend 300 years indicates the time period between 1700 to 2010, and so forth) with highest positive correlations between both population and cropland, and population and irrigation.

**Figure 3:**
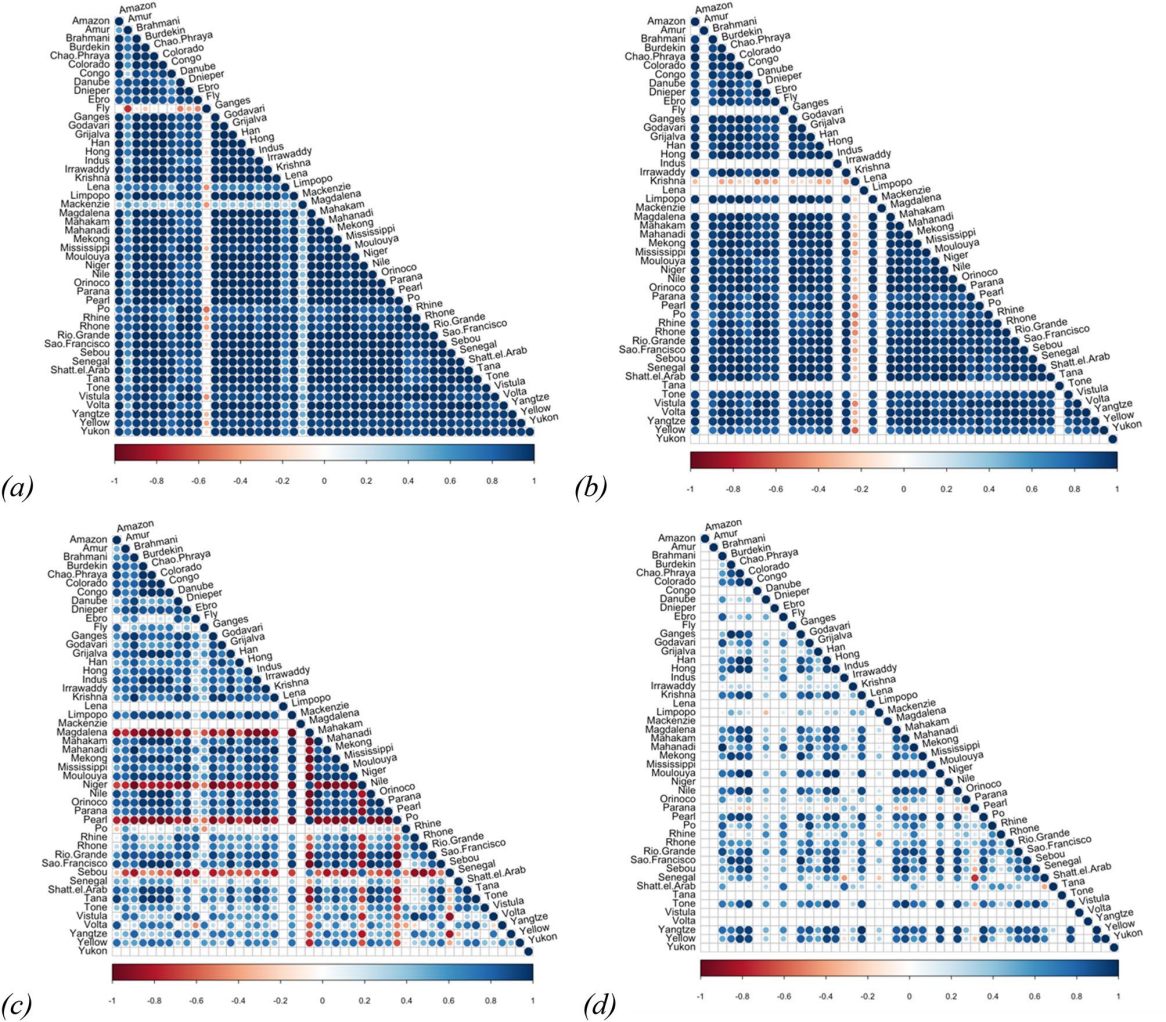
Pearson correlation between deltas, spatially averaged, over the past 310 years: (**a**) population (inh/km^2^), (**b**) urban (% of gridcell), (**c**) cropland (% of gridcell), and (**d**) irrigation (% of gridcell). Blank cells indicate deltas without agricultural land uses and therefore we could not calculate correlations.

According to our framework, locked-in states are linked to the development of population density and land use change over time. This is unsurprising as most of the development of infrastructure we report is in support of human population^2^; as substantial and faster population densities occur in deltas than elsewhere globally. On average, deltas have 10 times higher population densities (407.4 ± 1088 inh/km^2^) than the global average (30.4 ± 222.1inh/km^2^). Population growth in and outside deltas both follow exponential trajectories (Fig. 2), with an average delta’s population development of 8 inh/km^2^ per year. In the last 50 years, deltas exhibited substantial and faster population densities with an increase around 3 times that of the 1950s, while global population increased 2.3 times (see supplementary material for a detailed description of the development of population and land-use conversions in 48 deltas globally for the last 310 years). The deltas that have reached higher population densities are the Sebou, Pearl and Yangtze (ca. 2000–3000 inh/km^2^), followed by the Ganges, Govodari and Hong (ca. 1400–1600 inh/km^2^). In contrast, the Lena, Mackenzie and Amur deltas have less than 10 inh/km^2^; these deltas show a different population density development from all the others (Fig. 3a). Only the Fly delta shows a decrease in population density but numbers are extremely low. Of all the deltas, the Krishna is the only one showing an inverse development of urban areas (Fig. 3b). Average conversion to urban remains low (from 0.06% in 1700 to 2% in 2000, see Table SM1) throughout the last 310 years despite the high delta population densities.

## Transitions from natural ecosystems

Most deltas exhibit a cropland development that mostly follows an exponential growth at a lower rate than population density (Figure SM2 in the supplementary material). We find exceptions for cropland development in high latitude deltas (Lena and Mackenzie; Fig. 3c), which are still mainly pristine. The Magdalena, Niger, Pearl and Sebou show an inverse development in cropland compared to other deltas (Fig. 3c).

**Figure 4:**
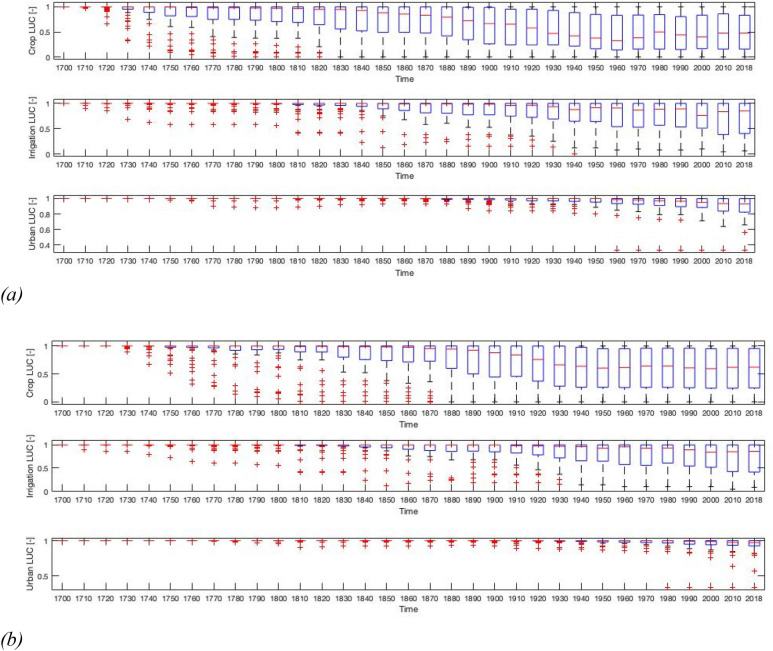
Boxplot of development of land-use change (LUC) for (**a**) 10% change and (**b**) 5% change from the original cell values in 1700. Calculation is done cell by cell. Boxplot box indicates the 25th and 75th percentiles, respectively. The whiskers extend to the most extreme data points not considered outliers, and the outliers are plotted individually using the ‘ + ’ symbol (outliers are values above and below two standard deviations from the mean).

The conversion to cropland is not constant over time, with changes of 10% steadily growing especially in 1880 and 1950 (Fig. 4a), while changes of 5% already occurred steadily since the 1800s.

The development of irrigation in deltas does not display the exponential characteristics of population density and cropland development (Figure SM3 in the supplementary material). The Limpopo, Parana and Senegal differ in their development of irrigation from five other deltas (red dots in Fig. 3d). Conversion to irrigation and urban land-uses occurs mostly since the 1850s, the area doubling after 1950s (Fig. 4). We find different patterns for cropland and irrigation development, as could be expected because irrigation requires additional investment and thus tends to appear later in the progression of land system development^33^. On average most cropland development took place before 1940, while urbanization and irrigation still slowly increase.

## Risk in locked-in and living deltas

Finally, we analyzed whether being historically locked-in makes deltas currently more at risk from a combination of hazardous events, unsuitable anthropogenic conditions and investment lags^2^. We expected that structural changes in natural components of deltas (i.e., river regularization, diking, water diversion, agricultural and urban development^11^) likely make them less resilient to hazardous events^29,34^, while structural changes to social components of deltas likely make them more vulnerable to anthropogenic conditioning^23^ and to investment lags^22^. These relations can be exacerbated by locked-in states, in particular as investment deficit is likely underestimated without knowledge of locked-in conditions. To test these hypotheses, we used the risk indices defined by Tessler et al*.*^2^ for our analysis, which define an average risk index as the overall risk in terms of risks due to hazardous events, exposure to antropogenic conditioning and vulnerability due to investment deficit that the delta experiences. Tessler et al*.*^2^ define the hazardous events index as the probability of fluvial or coastal flooding, the anthropogenic conditioning index as the expected number of people exposed to hazardous conditions, and the investment deficit index as a measure of the total investment (in infrastructure) needed for the delta to be resilient to the risk it is exposed to. In addition, we compared our locked-in delta classification with future relative sea level rise, to illustrate future challenges affecting these deltas. Relative sea level rises are dependent on local conditions of subsidence and regional estimates of sea level rise.

We find that the average risk index is significantly higher for locked-in deltas due to natural infrastructure changes, and is lowest for the social locked-in deltas (Table 1). This is logic as this index was developed for understanding natural risks^2^. However, living and locked-in deltas are equally at risk of hazardous events, i.e. the hazardous events index does not show significant differences between living and locked-in deltas. As hazardous events only focus on probability of fluvial or coastal flooding and not on social indicators this can be a logical explanation. Further, we find a trend towards a higher risk of future relative sea level rise for social locked-in deltas followed by living deltas.

**Table 1:** Delta state and risk categories.

Delta state	R	HEI	ACI	IDI	RSLR (mm/yr)
Living	0.09^a^	0.44 ^a^	0.38 ^a^	0.60 ^a^	6.7(5.3)^a,b,c^
Natural lock-in (crop)	0.13^a,b^	0.51 ^a^	0.55^b^	0.52 ^a,b^	6.4(2.8)^a,c^
Natural lock-in (irrigation)	0.14^b^	0.38 ^a^	0.50 ^b^	0.73^a^	4.1(0.6)^b^*
Social lock-in	0.07^a^	0.45 ^a^	0.49 ^b^	0.35^b^	8.7(5.9)^c^*

Different letters show statistically significant difference in means between delta categories (t-test.

R, Risk Index; HEI, Hazardous Events Index; ACI, Anthropogenic Conditioning Index; IDI, Investment Deficit Index; RSLR, relative sea level rise.

*p-value < 0.1 suggesting a trend, superscript letters indicate statistically different means at significance level of *p* < 0.05).

The differences in risks between delta states emerge in the exposure to antropogenic conditioning and vulnerability due to investment deficit. The anthropogenic conditioning index is clearly significantly higher in both natural and social locked-in deltas than in living deltas, which is understandable as this index uses population density that tends to be low in living deltas. The investment deficit index is clearly highest for natural lock-in due to irrigation development and for living deltas (Table 1). This is expected because Tessler et al*.*^2^ focused on investment deficit needed to respond to hazardous events, which is obviously higher for deltas lock-in due to irrigation development. We find that the investment deficit is also very high for living deltas, likely because those deltas are still undeveloped. On the other hand, the investment deficit is significantly lower in social lock-in deltas; this can be explained by the way the investment deficit is defined^2^, and also because we include the historical analysis of social development which has not been previously included in risk assessments. Nonetheless, we find a significant positive effect of the correlation between population and cropland over the last 50 years on overall risk (R), while a significant negative effect on Investment Deficit Index (Figure SM4; Table SM3). We also conducted a Principal Component Analysis which revealed that for locked-in due to cropland in the last 50 years it is mostly associated with IDI, while for the full 300 years is associated with overall risk (but a weak relationship; Figure SM5). These results suggest that while currently at risk lock-in deltas are of urgent priority, these priorities will shift in the near future especially in deltas lock-in due to social infrastructure. Adding the historical perspective to risk assessments provides new insights in the coupled dynamics of deltas social-ecological systems and also highlights the need to better include social aspects. Therefore, it is important to devise different actions depending on why the delta is locked-in. Finally, we find that the current living deltas will likely experience unprecedented risks, and therefore more attention and proactive action is needed to prevent them from reaching a locked-in state and exacerbate the risks they may face. For instance, we think that wetland restoration and biodiversity protection can go hand in hand as a proactive action to prevent deltas to move into locked-in states.

## Framework for locked-in deltas

Our framework highlights the importance of understanding to what extent the development of combined pressures and risks has led delta systems to become locked-in. Our results are in line with previous research that identified deltas as locked-in in Europe (the Dutch Zeeland delta^13^; the Rhine^19^), and elsewhere (Yangtze^19^; Niger^18^). However, our results were mismatched for the Mekong^16^ and the Nile delta^19^. The lock-in for the Mekong delta is attributed to surplus rice exports now threatened by rising sea levels and salinity intrusion^35^, while the lock-in for the Nile is attributed to dam development^19^. Expanding our framework to include land-use change in the watershed would, we believe, allow us to detect the Nile lock-in. However, the indirect land-use changes driving the Mekong lock-in are more difficult to account for in such a simple framework, a known limitation to indirect land-use change analyses^36–38^. While we believe that for the historical time period these indirect land-use changes may have not yet had much of an effect, they will grow in importance in future. Delta regions produce a large amount of food for export (similar processes are found in the Sacramento-San Joaquin inverse delta in California not analyzed here;^14^). It could be expected that the deltas exhibiting exponential cropland and irrigation development will be more likely to have indirect land-use changes due to export of agricultural goods^33^. We do not expect that these mismatches are related to the use of HYDE, in comparison to other databases, as while estimates of land use intensity differ^33^.

## Living or locked-in deltas in the anthropocene

Deltas have historically dealt with many of the multisectorial challenges to future sustainable development. Our framework builds on those challenges to identify locked-in states, i.e. when systems are unable or too costly to recover, and find that half of the deltas are yet to be locked-in (23 out of 48) due to land-use changes. These deltas are mostly tropical, with some northern hemisphere deltas, excepting European ones. However, other characteristics could determine whether a delta is locked-in or not. For example, Day et al*.*^4^ used geomorphic, ecological and economic characteristics of deltas to categorize them in terms of their sustainability. When compared with our land use based approach, we observe that for instance, within our living deltas, Day et al*.*^4^ classifies the Amazon as highly sustainable, while the Colorado is deemed highly unsustainable due to changes in freshwater drainage. The advantage is the long period over which our framework can be applied during the Anthropocene.

Deltas are quintessential case studies to assess the degree to which these multi-sectorial challenges of growing population and land use change over limited areas can and have been addressed. Currently most deltas are locked-in due to changes in natural infrastructure but it is becoming clear that social lock-ins also play a role now and in the future, which encourages the analysis of deltas as coupled social-ecological systems. Renaud et al*.*^11^ analyzed five major deltas with high human impacts, all classified in our framework as locked-in, for the potential of collapse by passing tipping points. We show that a simple framework allow us to identify potential locked-in pathways in deltas and when they emerge. Our framework shows how a system moves from a living delta, to a locked-in delta by engineering natural infrastructure and finally to changes in social infrastructure and institutions. This framework could be expanded to include other mechanisms through which locked-in paths can emerge, such as upstream land use changes, effects of long time protection for biodiversity and potentially include drivers of indirect land use change. This is fundamental to assess whether the most at risk deltas are or are not able to deal with such challenges due to path dependencies from past decisions. We find promising results that most deltas are not locked-in and therefore have the capacity to respond to pressures and risks.

## Methods

### Historical reconstruction

We selected the same 48 deltas as Tessler et al*.*^2^ for our analysis. These deltas were selected because they represent some of the largest deltas globally, with an extensive body of knowledge and holding large population sizes. We used the HYDE data base—History Database of the Global Environment (https://themasites.pbl.nl/tridion/en/themasites/hyde/) to obtain gridded estimates of land use and population density at 5 min resolution, about 10 × 10 km. HYDE is an internally consistent combination of historical population estimates and allocation algorithms that provide time-dependent and culturally adjusted values for land use. Land use categories include irrigated and rain-fed cropland and rice, and grazing land, split into pasture and rangeland. Population is represented by maps of total population density and built-up area. The period covered is 10,000 Before Common Era (BCE) to current. We chose to analyze the Anthropocene period, i.e., 1700 until 2010. Population data for 1950 to current are from United Nations World Populations Prospects (UN, 2008). Pre-1950 population data are from historical estimates from various sources (see for details^31,32^). These subnational population data are downscaled to 5 min resolution grid cells by using the spatial population patterns from LandScan (LandScan 2014) at 1 km resolution, consisting of urban and rural patterns. From these downscaled patterns of human occupation, built-up area per 5 min grid cell is computed. Rain-fed and irrigated croplands are also extracted from HYDE. From 1960 to 2015 CE, mainly FAO data (FAO, 2015) are used for both land-use types. Pre-1960 cropland area was derived from a combination of population and per capita land use estimates, and modelled back in time and tuned with specific historical data as shown in Klein Goldewijk et al*.*^31,32^. Irrigated land before 1960 is from Siebert et al*.*^39^. We extracted population density (inhabitants per km^2^) for the grid cells that covered each delta, as well as the proportion of the grid cell covered by cropland, irrigation and urban area. We only use urban land use to demonstrate delta development; the remaining land use types are used to test our framework to assess delta locked-in.

### Delta development

We determined whether historical development of population, cropland and irrigation in deltas were similar to the global average. Since deltas were always attractors of population and subsequent land use, on average the growth rates of these dimensions should be higher than global averages^3^. Further, given the geographical patterns to population development, deltas at different latitudes may experience different historical development paths. First, we plotted population density and land uses (cropland, irrigation and urban) over time per delta. For each curve, we estimated line of best fit to assess whether there were generalities in both population density and land use development pathways. Second, we calculated the growth rate of population density and land use intensity per delta over the whole time period (1700-current) and then with time slices of 50 years less, e.g. 1750-current etc., and repeated these calculations per latitude and for non-delta areas as well. Therefore we can analyze if there is a difference in growth rate between deltas and non-deltas and if growth rates and differences between deltas and non-deltas have changed over time. For non-delta regions we have calculated average values per 1 degree latitude. For delta-regions we have analyzed average values over 5 degree latitude regions to smoothen the signal since there are relatively fewer cells in deltas than at other latitudes. Third, we wanted to measure development of land uses per cell over time, i.e., how the cell fraction covered by a given land use developed since 1700. To do so, we analyzed if a 5 min cell in the delta changed its land use in comparison to the initial land use/cover of that cell in 1700; we calculated for every time slice if a cell has changed their with a magnitude of 5 or 10% of the cell. We use the data from all the 48 deltas to plot boxplots where a value of 1 means that there is no change from the 1700 land use/cover. A value of 0 will mean that all cells have a change of at least 5 or 10%. For example, for cropland and irrigation, the cells correspond to 5 min cells (at the equator 10 × 10 km), while urban area is measured in km^2^. So, for urban area we consider a change of 10 km^2^, roughly the same as a 10% change in cover for a 5 min cell.

### Detecting lock-in

We posit that:

- if a delta is not locked-in, i.e., a living delta, there should be a positive correlation between development of population density and cropland because enough land remains for both to develop. Also, pristine deltas with low population densities (< 1 inhabitant/km^2^) are seen as living deltas.
- if a delta is locked-in due to engineered natural infrastructure, there should be a shift (b1) from a positive to no correlation between population density and cropland during the Anthropocene or (b2) from a positive to no correlation between population density and irrigation during the Anthropocene.
- if a delta is locked-in due to social infrastructure, then correlation between population and agricultural land uses (either cropland or irrigation) becomes negative during the Anthropocene, as then population and agricultural land uses develop at odds.

Note that deltas tend to develop from (a) to (b) and then to (c). Given our analyses started at the beginning of the Anthropocene, some deltas (mostly European) were already in state (b).

We used the Pearson correlation coefficient, with r > 0.5 as a positive correlation and r < − 0.5 as a negative correlation. False positives could occur when a downward population trend positively correlated with a downward trend in cropland and irrigation. However, we find no such case, most deltas showing an upward and even exponential population growth. The land system development sequence should therefore start with deltas that are living, followed by those showing engineered natural system locked-in, and finally those locked-in due to social infrastructure.

## Supplementary information

Supplementary Information

## Data Availability

FAO: FAOSTAT, Food and Agriculture Organization of the United Nations, Rome, Italy, available at: https://www.fao.org, last access: June 2015. LandScan: Landscan Global Population Database, The 2012 Revision, Oak Ridge National Laboratory, Oak Ridge, Tennessee, available at: https://www.ornl.gov/landscan (last access: January 2015). UN 2008. World Population Prospects, The 2007 Revision, United Nations Population Division, New York.
