# Supplementary material for: Locked-in and living delta pathways in the Anthropocene
Source: Sci Rep. 2020 Nov 11;10:19598. doi: 10.1038/s41598-020-76304-x (PMC7659346; doi:10.1038/s41598-020-76304-x)
Supplement: Supplementary file 1 — Supplementary Information [file 41598_2020_76304_MOESM1_ESM.docx]

**Title**: Locked-in and living delta pathways in the Anthropocene

**Authors:** Maria J. Santos^1^ and Stefan C. Dekker^2^

**Supplementary material**

Development of population and land use conversions in 48 deltas globally over the last 310 years.

**Table SM1.** Mean and standard deviation of population, cropland, irrigation and urbanization growth in analyzed deltas. We find an increasing standard deviation in land use change per delta over time. Variability in global population is much higher than in deltas.

|  | Population density  (inh/km^2^) | | Cropland  (% of gridcell) | | Irrigation  (% of gridcell) | | Urbanization  (% of gridcell) | |
| --- | --- | --- | --- | --- | --- | --- | --- | --- |
| Year | Deltas | All | Deltas | All | Deltas | All | Deltas | All |
| 1700 | 27.6+/-28.7 | 2.9+/-12.5 | 10.5+/-8.0 | 1.4+/-5.0 | 0.80+/-3.1 | 0.02+/-0.74 | 0.06+/-0.37 | 0.00+/-0.13 |
| 1750 | 35.1+/-37.4 | 3.6+/-15.6 | 11.5+/-7.7 | 1.7+/-5.3 | 0.91+/3.7 | 0.03+/-0.90 | 0.10+/-0.62 | 0.01+/-0.17 |
| 1800 | 45.8+/-58.0 | 4.6+/-19.9 | 12.3+/-7.4 | 2.1+/-5.7 | 1.05+/-4.5 | 0.04+/-1.06 | 0.15+/-1.1 | 0.01+/-0.27 |
| 1850 | 64.7+/-100.1 | 6.2+/-29.6 | 14.5+/-7.7 | 2.8+/-6.8 | 2.61+/-7.2 | 0.13+/-1.90 | 0.24+/-2.1 | 0.02+/-0.37 |
| 1900 | 85.0+/-137.6 | 8.3+/-50.7 | 17.3+/-7.9 | 4.3+/-8.1 | 3.90+/-7.8 | 0.22+/-2.48 | 0.44+/-2.9 | 0.05+/-0.59 |
| 1950 | 137.6+/-272 | 13.0+/-81.2 | 20.9+/-8.6 | 6.4+/-8.9 | 5.98+/-9.7 | 0.50+/-3.9 | 0.79+/-5.6 | 0.10+/-1.21 |
| 2000 | 407.4+/-1088 | 30.4+/-222 | 24.4+/-9.5 | 7.8+/-9.7 | 11.3+/-12.4 | 1.08+/-5.7 | 2.1+/-7.3 | 0.25+/-2.21 |

**Table SM2.** Exponential equation and goodness of fit (coefficient of determination) for population density, cropland and irrigation over the last 300 years in the 48 deltas. Blank means no significant fit (p>0.05).

|  | Population | | Cropland | | | Irrigation | | |
| --- | --- | --- | --- | --- | --- | --- | --- | --- |
|  | Equation | R² | Equation | R² | Equation | | R² |  |
| Amazon | y = 1 x10^-18^e^0.022x^ | 0.98 | y = 4 x10^-23^e^0.0258x^ | 0.95 |  | |  |  |
| Amur | y = 0.002e^0.0037x^ | 0.90 | ---- | --- |  | |  |  |
| Brahmani | y = 0.004e^0.0056x^ | 0.86 | y = 0.1e^0.0032x^ | 0.92 |  | |  |  |
| Burdekin | y = 8 x10^-15^e^0.0173x^ | 0.89 | ---- | --- |  | |  |  |
| Chao Phraya | y = 6 x10^-8^e^0.0112x^ | 0.86 | y = 6 x10^-6^e^0.0077x^ | 0.76 | y = 8 x10^-21^e^0.0246x^ | | 0.91 |  |
| Colorado | y = 2 x10^-14^e^0.0174x^ | 0.89 | y = 4 x10^-10^e^0.0121x^ | 0.94 | y = 3 x10^-10^e^0.0122x^ | | 0.93 |  |
| Congo | y = 6 x10^-9^e^0.0109x^ | 0.60 | y = 0.0001e^0.0049x^ | 0.76 |  | |  |  |
| Danube | y = 2 x10^-6^e^0.0087x^ | 0.99 | y = 0.11e^0.0025x^ | 0.82 |  | |  |  |
| Dnieper | y = 1 x10^-9^e^0.0127x^ | 0.99 | y = 3 x10^-5^e^0.0067x^ | 0.96 |  | |  |  |
| Ebro | y = 0.047e^0.0039x^ | 0.94 | y = 5.58e^0.0009x^ | 0.51 | y = 0.0001e^0.0061x^ | | 0.70 |  |
| Fly | y = 161.7e^-0.004x^ | 0.58 | y = 0.18e^0.0009x^ | 0.18 |  | |  |  |
| Ganges | y = 0.002e^0.0064x^ | 0.82 | y = 3.9e^0.0013x^ | 0.96 |  | |  |  |
| Godavari | y = 0.0003e^0.0072x^ | 0.89 | y = 1.03e^0.0019x^ | 0.71 | y = 0.0005e^0.0058x^ | | 0.88 |  |
| Grijalva | y = 6 x10^-10^e^0.0125x^ | 0.86 | y = 1 x10^-6^e^0.0085x^ | 0.98 |  | |  |  |
| Han | y = 2 x10^-5^e^0.0087x^ | 0.86 | y = 2.6e^0.0013x^ | 0.63 |  | |  |  |
| Hong | y = 7e-07e^0.0104x^ | 0.96 | y = 0.1e^0.0029x^ | 0.92 |  | |  |  |
| Indus | y = 0.13e^0.0029x^ | 0.31 | y = 0.0002e^0.006x^ | 0.84 |  | |  |  |
| Irrawaddy | y = 2 x10^-5^e^0.0081x^ | 0.92 | y = 0.0014e^0.0051x^ | 0.89 |  | |  |  |
| Krishna | y = 0.008e^0.0052x^ | 0.84 | y = 0.2e^0.0028x^ | 0.84 | y = 4 x10^-7^e^0.0093x^ | | 0.92 |  |
| Lena | y = 1 x10^-5^e^0.0033x^ | 0.87 | ---- | --- |  | |  |  |
| Limpopo | y = 9 x10^-8^e^0.0104x^ | 0.96 | y = 0.003e^0.0042x^ | 0.98 | y = 0.3e^0.0007x^ | | 0.003 |  |
| Mackenzie | y = 1 x10^-16^e^0.0172x^ | 0.93 | ---- | --- |  | |  |  |
| Magdalena | y = 9 x10^-11^e^0.0143x^ | 0.92 | y = 13395e^-0.003x^ | 0.75 |  | |  |  |
| Mahakam | y = 4 x10^-16^e^0.02x^ | 0.97 | y = 5 x10^-9^e^0.0113x^ | 0.94 |  | |  |  |
| Mahanadi | y = 0.0003e^0.0072x^ | 0.91 | y = 0.05e^0.0036x^ | 0.89 |  | |  |  |
| Mekong | y = 3 x10^-6^e^0.0091x^ | 0.93 | y = 4 x10^-5^e^0.0069x^ | 0.97 | y = 2 x10^-13^e^0.016x^ | | 0.97 |  |
| Mississippi | y = 1 x10^-17^e^0.022x^ | 0.98 | y = 4 x10^-13^e^0.016x^ | 0.91 |  | |  |  |
| Moulouya | y = 3 x10^-9^e^0.0123x^ | 0.97 | y = 9 x10^-5^e^0.0066x^ | 0.99 |  | |  |  |
| Niger | y = 0.006e^0.0049x^ | 0.66 | y = 14978e^-0.004x^ | 0.96 |  | |  |  |
| Nile | y = 1 x10^-6^e^0.0101x^ | 0.83 | y = 0.0004e^0.0056x^ | 0.73 | y = 0.0004e^0.0056x^ | | 0.73 |  |
| Orinoco | y = 7 x10^-10^e^0.011x^ | 0.87 | y = 6 x10^-8^e^0.0083x^ | 0.90 |  | |  |  |
| Parana | y = 4 x10^-17^e^0.0208x^ | 0.91 | y = 1 x10^-8^e^0.0102x^ | 0.95 |  | |  |  |
| Pearl | y = 1 x10^-7^e^0.0117x^ | 0.95 | y = 1577.5e^-0.002x^ | 0.81 | y = 1 x10^-8^e^0.0103x^ | | 0.95 |  |
| Po | y = 0.06e^0.0037x^ | 0.97 | y = 15.9e^0.0005x^ | 0.13 |  | |  |  |
| Rhine | y = 0.0002e^0.0075x^ | 0.93 | y = 0.1e^0.0024x^ | 0.72 |  | |  |  |
| Rhone | y = 0.03e^0.0042x^ | 0.92 | y = 1.9e^0.0009x^ | 0.62 | y = 0.009e^0.0033x^ | | 0.63 |  |
| Rio Grande | y = 7 x10^-15^e^0.0186x^ | 0.96 | y = 2 x10^-12^e^0.0154x^ | 0.94 |  | |  |  |
| São Francisco | y = 3 x10^-14^e^0.0178x^ | 0.99 | y = 4x10^-16^e^0.0191x^ | 0.98 |  | |  |  |
| Sebou | y = 9 x10^-11^e^0.0152x^ | 0.98 | y = 633.9e^-0.002x^ | 0.72 |  | |  |  |
| Senegal | y = 1 x10^-5^e^0.0074x^ | 0.70 | y = 1.7e^0.0003x^ | 0.03 | y = 0.7e^0.0005x^ | | 0.06 |  |
| Shatt-el-Arab | y = 1 x10^-8^e^0.011x^ | 0.94 | y = 0.2e^0.0016x^ | 0.41 |  | |  |  |
| Tana | y = 0.001e^0.0042x^ | 0.53 | y = 0.01e^0.0025x^ | 0.42 |  | |  |  |
| Tone | y = 0.7e^0.003x^ | 0.60 | y = 3.7e^0.001x^ | 0.20 | y = 0.003e^0.0046x^ | | 0.78 |  |
| Vistula | y = 9 x10^-6^e^0.0087x^ | 0.99 | y = 0.4e^0.0023x^ | 0.73 |  | |  |  |
| Volta | y = 0.0001e^0.007x^ | 0.68 | y = 5.3e^0.0004x^ | 0.02 |  | |  |  |
| Yangtze | y = 0.0001e^0.008x^ | 0.94 | y = 0.38e^0.0023x^ | 0.62 | y = 7 x10^-9^e^0.0112x^ | | 0.96 |  |
| Yellow | y = 0.0125e^0.0047x^ | 0.86 | y = 6.9e^0.0006x^ | 0.37 |  | |  |  |
| Yukon | y = 4 x10^-7^e^0.0066x^ | 0.74 | ---- | --- |  | |  |  |


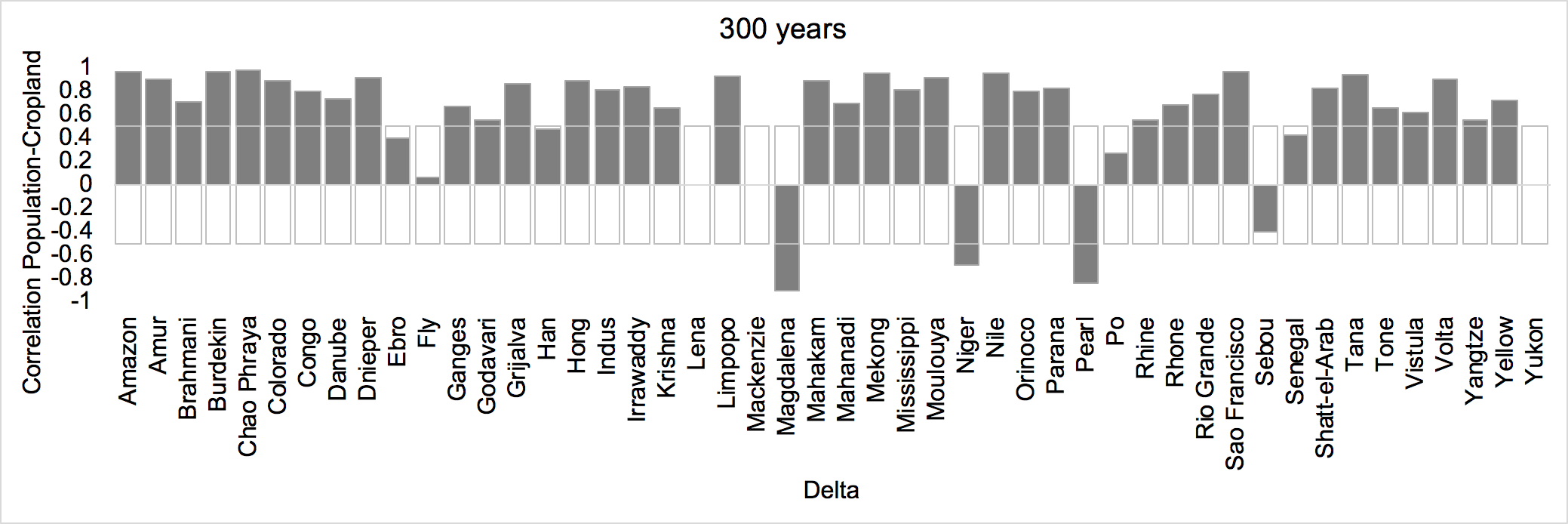


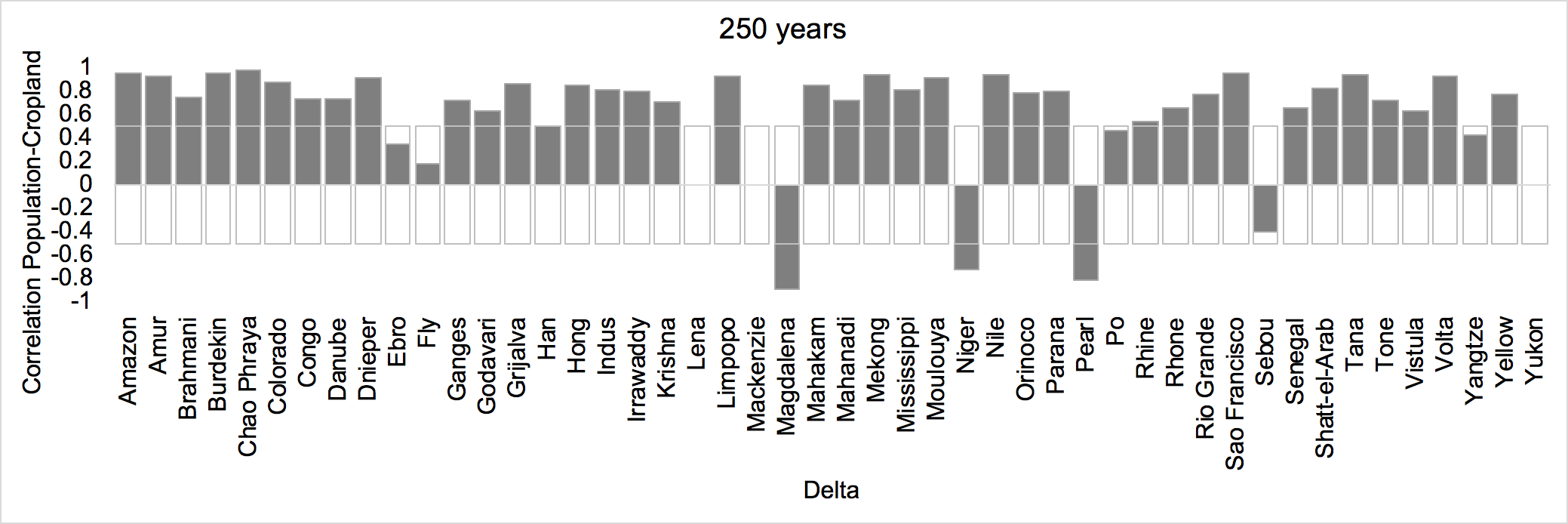


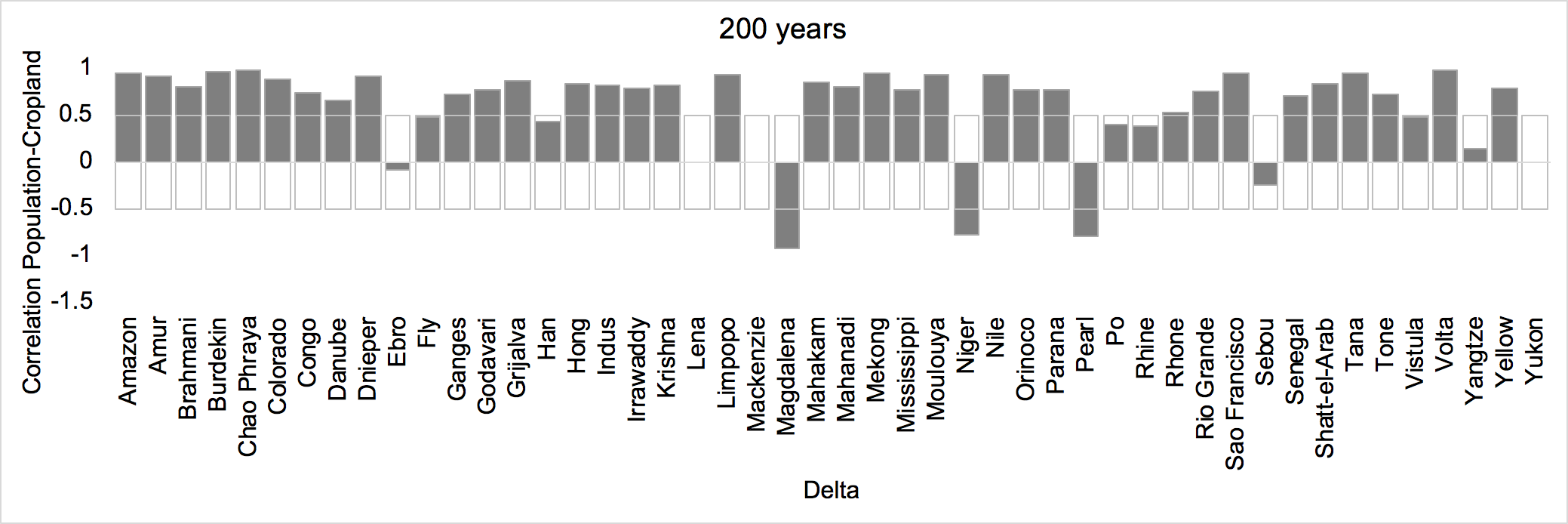


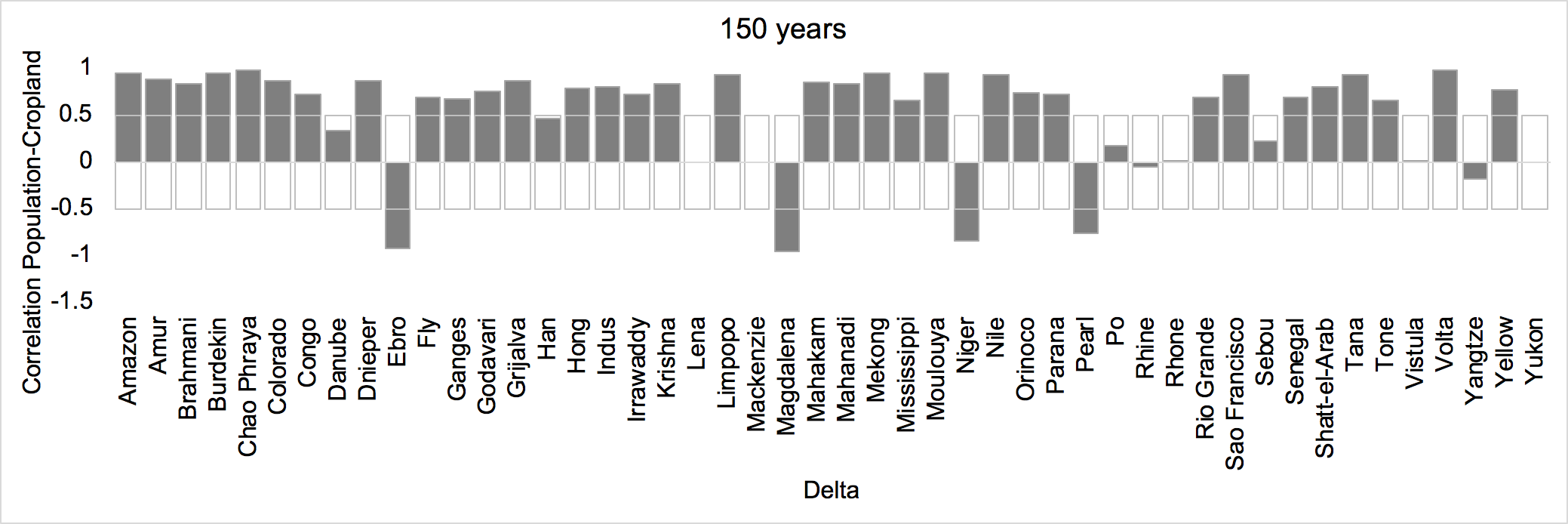


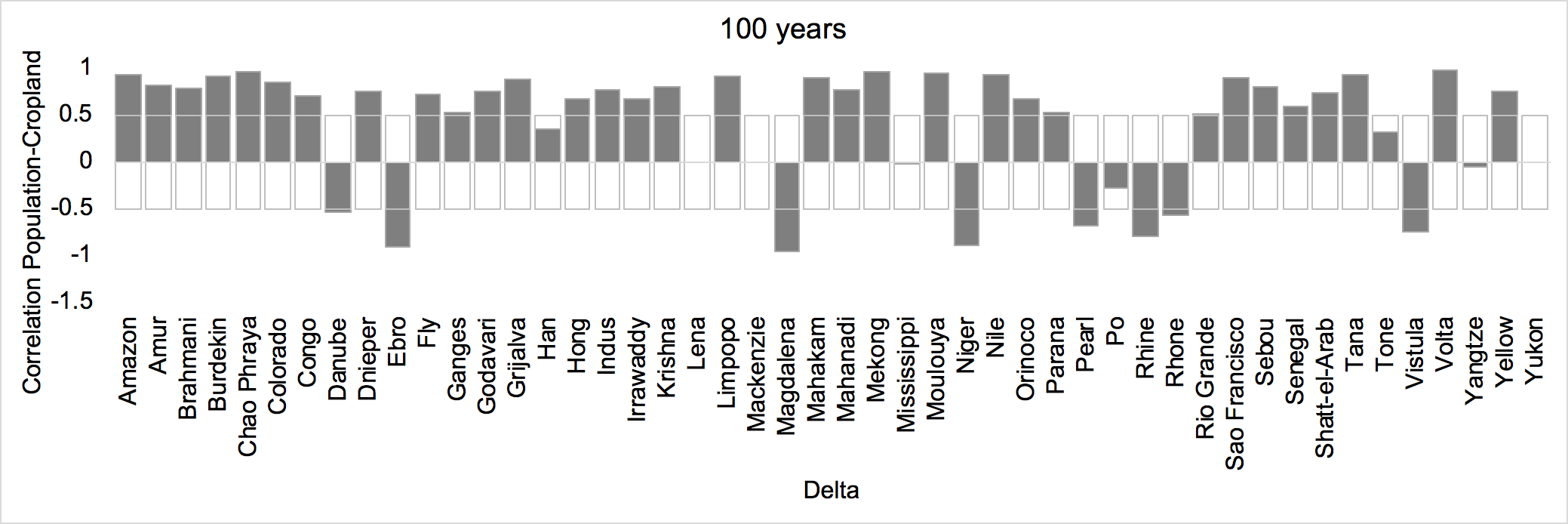


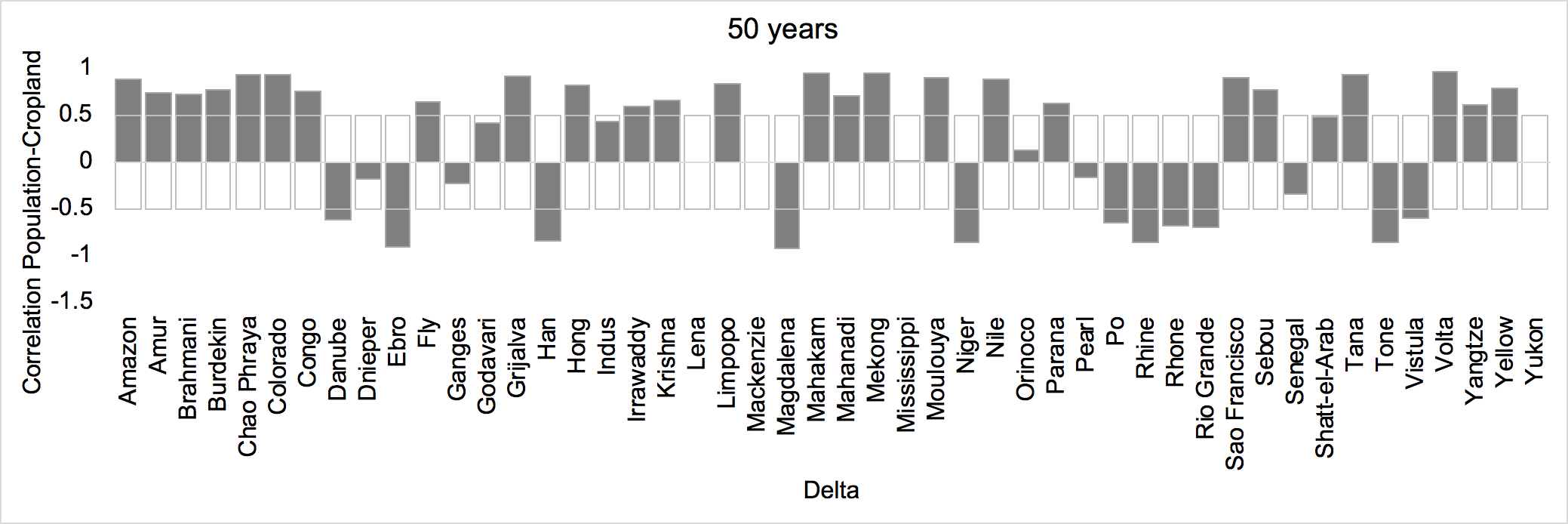


**Figure SM1.** Development of lock-in due to decoupling of the correlation between population and cropland development. Most lock-ins emerge in the last 100 years, with the exception of European deltas, which have a longer history of land-use.

**Figure SM2:** Cropland development in the analyzed deltas. In general cropland development is more linear than population development, the highest values found for the Brahmani and Krishna. The Lena, Mackenzie and Yukon do not show any cropland development, while the Magdalena and Niger deltas show negative cropland development over the last 300 years. Some deltas showed no significant trend (Table SM2).

**Figure SM3:** Irrigation development in the analyzed deltas. In general irrigation development is less frequent than agriculture across deltas, with highest values found for Chao Phraya, Ganges, Godovari, Mahanadi, Mekong, Moulouya, Tone and Yangtze. Some deltas showed no significant trend (Table SM2).

We analyzed whether deltas with different levels of lock in would show differences in their current overall risk (R), as well as differences in the Investment Deficit Index (IDI), Anthropogenic Conditioning Index (ACI), Hazardous Events Index (HEI) and Relative Sea Level Rise (RSLR).

In Figure **SM4.** we show the box plots of the Tessler risk indices and RSLR by delta lock-in state. We can see that Risk is lower for social lock in and living deltas than other lock-in deltas, and IDI is higher for lock in deltas by irrigation. Finally, living deltas tended to have a lower ACI while no differences can be reported for HEI across delta types. These results are in line with those reported in table 1 in the main text.

**Figure SM4.** Box plots of delta types (locked in and living deltas) for relative sea level rise, overall risk (R), Investment Deficit Index (IDI), Anthropogenic Conditioning Index (ACI), and Hazardous Events Index (HEI). We find

We also tested the hypothesis on whether the correlation between population and cropland and irrigation would predict current risk status, both for the strength of the correlation but also whether this relationship would vary when considering the correlation over 300 years and over the last 50years. We find a significant positive effect of the correlation between population and cropland over the last 50 years on overall risk (R), while a significant negative effect on Investment Deficit Index. This suggests that indeed low correlations, i.e. delta states closer to recent lock-in have higher Investment Deficit Index values.

**Table SM3.** Generalized linear models for the correlation between population density and crop and irrigation over 300 years and 50 years and the risk indices.

|  | Crop300 | Crop50 | Irrigation300 | Irrigation50 |
| --- | --- | --- | --- | --- |
| R | Estimate=0.006  T test=0.289  P-value=0.77 | **Estimate=0.027**  **T test=1.96**  **P-value=0.05** | Estimate=-0.008  T test=-0.215  P-value=0.83 | Estimate=0.01  T test=0.526  P-value=0.6 |
| ACI | Estimate=-0.06  T test=-1.138  P-value=0.26 | Estimate=-0.05  T test=-1.532  P-value=0.133 | Estimate=0.037  T test=0.439  P-value=0.66 | Estimate=-0.04  T test=-0.77  P-value=0.45 |
| HEI | Estimate=0.05  T test=0.958  P-value=0.34 | Estimate=-0.01  T test=-0.274  P-value=0.78 | Estimate=0.09  T test=0.94  P-value=0.35 | Estimate=0.048  T test=0.88  P-value=0.39 |
| IDI | Estimate=0.02  T test=0.3  P-value=0.77 | **Estimate=0.168**  **T test=3.853**  **P-value=0.0004** | Estimate=-0.12  T test=-1.032  P-value=0.31 | Estimate=0.05  T test=0.838  P-value=0.4 |

In addition, we conducted two Principal Component Analyses to assess how well do the correlations between population and cropland and irrigation link to the indicators of delta risk. We find that the first three PCAs explain more than 75% of the variation in the data (Table SM3).

**Table SM4**. Principal Component Axes for the PCAs for the correlation between population density and crop over 300 years (Crop 300y) and 50 years (Crop50y) and Irrigation over 300 years (Irrigation300y) and 50 years (Irrigation50y).

|  | Crop300y | Crop50y | Irrigation300y | Irrigation50y |
| --- | --- | --- | --- | --- |
| PCA1 | 0.315 | 0.321 | 0.355 | 0.323 |
| PCA2 | 0.244 | 0.299 | 0.258 | 0.244 |
| PCA3 | 0.188 | 0.155 | 0.150 | 0.176 |
| Cumulative | 0.747 | 0.775 | 0.763 | 0.743 |

Further, when plotting the first two components for each of the PCAs we can observe that the PCAs for the later 50years correlations are much better at splitting the delta types for cropland, and similar distribution of delta types occurs for lock-ins due to irrigation. These groupings are also associated with different risk indicators. For locked-in due to cropland, in the last 50years is mostly associated with IDI, while for the full 300 years is associated with overall risk (but a weak relationship); these results are in line with the results of the generalized linear model. On the other hand, for irrigation, the last 50years are mostly associated with ACI while the full 300years are associated with HEI, but the clustering is not robust and not supported by the glm model results (Figure SM5).


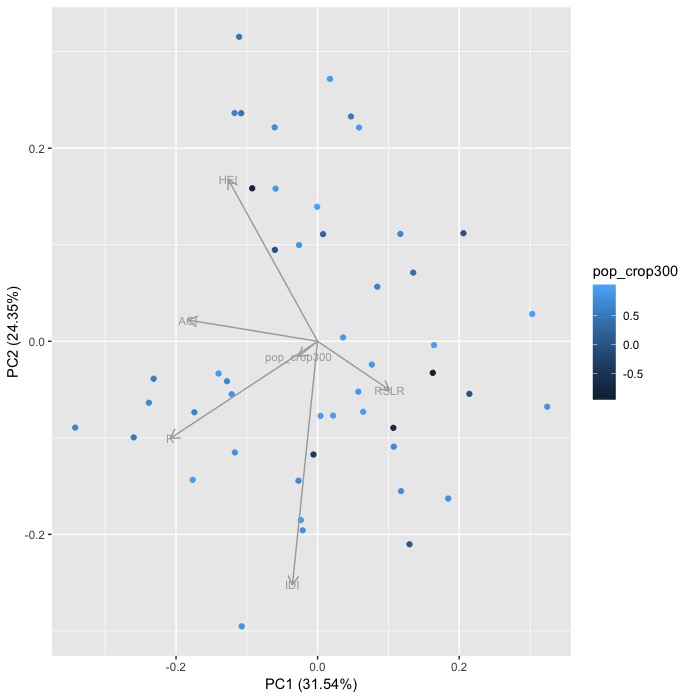

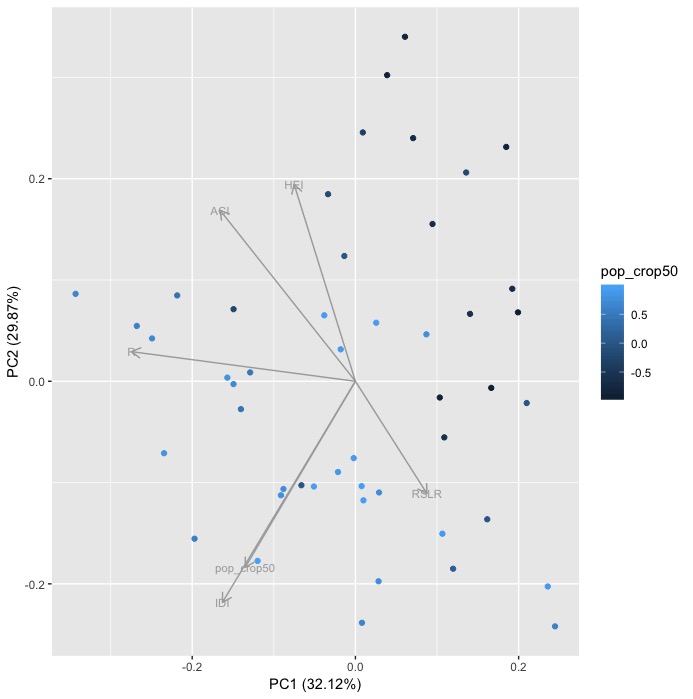

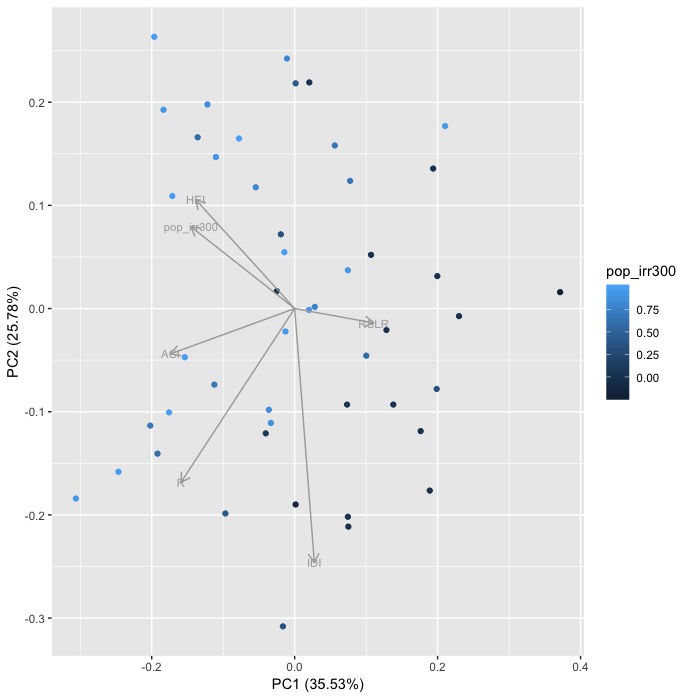

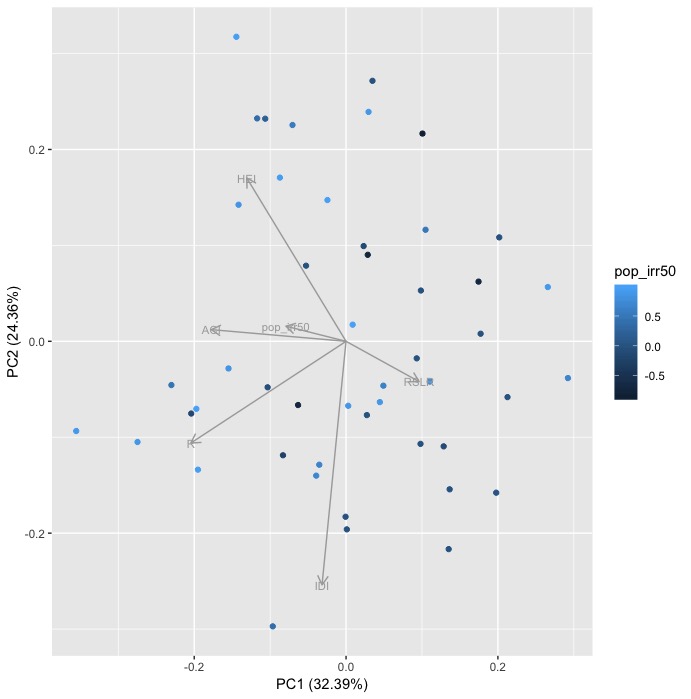


**Figure SM5**. PCA plots displaying the first two principal components for the correlation between population density and crop over 300 years (Crop 300y) and 50 years (Crop50y) and Irrigation over 300 years (Irrigation300y) and 50 years (Irrigation50y). Vectors represent the different risk indices.
